# Scoring Divergent Thinking Tests by Computer With a Semantics-Based Algorithm

**DOI:** 10.5964/ejop.v12i2.1127

**Published:** 2016-05-31

**Authors:** Kenes Beketayev, Mark A. Runco

**Affiliations:** a Nazarbayev University, Astana, Kazakhstan; b Sparcit, Inc., Davis, CA, USA; c American Institute of Behavioral Research & Technology, Vista, CA, USA; Aalborg University, Aalborg, Denmark

**Keywords:** Divergent thinking, assessing creativity, creativity test, flexibility, ideas, originality, semantic networks, associative networks, computer creativity, ideational fluency

## Abstract

Divergent thinking (DT) tests are useful for the assessment of creative potentials. This article reports the semantics-based algorithmic (SBA) method for assessing DT. This algorithm is fully automated: Examinees receive DT questions on a computer or mobile device and their ideas are immediately compared with norms and semantic networks. This investigation compared the scores generated by the SBA method with the traditional methods of scoring DT (i.e., fluency, originality, and flexibility). Data were collected from 250 examinees using the “Many Uses Test” of DT. The most important finding involved the flexibility scores from both scoring methods. This was critical because semantic networks are based on conceptual structures, and thus a high SBA score should be highly correlated with the traditional flexibility score from DT tests. Results confirmed this correlation (r = .74). This supports the use of algorithmic scoring of DT. The nearly-immediate computation time required by SBA method may make it the method of choice, especially when it comes to moderate- and large-scale DT assessment investigations. Correlations between SBA scores and GPA were insignificant, providing evidence of the discriminant and construct validity of SBA scores. Limitations of the present study and directions for future research are offered.

Divergent thinking tests are probably the most commonly-used assessment for creative potential. Divergent thinking (DT) is not synonymous with creativity but tests of DT do provide useful information about creative potential. In particular, they provide scores for *ideational fluency*, which represents the number of ideas an individual gives, *ideational flexibility*, which represents the number of different conceptual categories used by the individual, and *ideational originality*, which represents the statistical infrequency or uniqueness of ideas. Originality is especially important because it is a part of the standard definition of creativity (Runco & Jaeger, 2012). Ideational flexibility is also extremely important (Runco, 1986). It allows an individual to avoid ruts and routines when solving problems. This in turn contributes to creative problem solving but is also related to adaptability and to the capacity to shift perspectives when solving a problem.

There are different ways to score divergent thinking tests (Acar & Runco, 2014; Hocevar, 1979; Hocevar & Bachelor, 1989; Hocevar & Michael, 1979; Milgram, 1990; Runco, Okuda, & Thurston, 1987; Torrance, 1995). Some of the most recent methods for the scoring of divergent thinking tests employ computers. Acar and Runco (2014), for example, gave divergent thinking tests to a group of individuals via computer and then scored these tests using three semantic networks. They were especially interested in *associative distance*. This represents how far (in conceptual space) one idea given by one individual was from other ideas given by the same individual. Acar and Runco reported that associative distance could be reliably measured and that it was statistically correlated with an originality scale from a creative attitudes and values survey. By far the most comprehensive computer-based method for scoring divergent thinking tests is the semantic-based algorithm (SBA) developed by SparcIt (http://cit.sparcit.com). It uses 12 semantic networks when coding ideas and continually improves over time as it processes more data.

Semantic networks quantify relationships between concepts (Sowa, 1991). One variation of a semantic network is an *association network* wherein semantic relations are interpreted as connections between words and concepts in natural language. These form lexical neighborhoods within an association network (Rapp & Samuel, 2002; Ruge, 1992). The association networks used in the previous research (Acar & Runco, 2014) were the Word Association Network (WAN), developed by Rotmistrov (http://wordassociations.net/), WordNet (http://wordnet.princeton.edu), and IdeaFisher (http://throughtrod.com/). The 12 semantic networks used in the present research were constructed using a combination of associative networks and semantic co-occurence distances. Utilizing 12 semantic networks has an advantage over using fewer (such as the 3 in Acar & Runco, 2014), namely generalizability. Simplifying some, results form 12 networks are more indicative of natural language (and associations) than are results from 3 networks. It is analogous to having a larger normative sample when interpreting some test result or score.

The present study was conducted to determine how the indices produced by the SBA are related to the traditional scores (fluency, originality, flexibility) from divergent thinking tests. Information about these relationships is of enormous practical and psychometric interest. The computer program is likely to generate highly reliable scores, for instance, given that no human judgment is required. It is also highly cost efficient. But these computer-generated scores may not tell us everything about ideas and divergent thinking. They are, after all, based entirely on semantic networks. It is possible that the computer algorithm will provide reliable scores, but scores which are unrelated to those resulting from the standard manual system. This is of course an empirical question—and the primary justification for the present study.

Given the semantic basis of the SBA method, a strong correlation was expected between the SBA flexibility score and the flexibility index of the traditional scoring method. Indeed, that correlation should be higher than a correlation of flexibility with fluency or originality. A second expectation was that the SBA method would generate scores that would be unrelated to GPA. This follows from the theory that divergent thinking is unrelated to convergent thinking, and that GPA and other measures of traditional intelligence are highly dependent on convergent thinking (Acar & Runco, 2015; Guilford, 1968; Kim, 2005; Runco & Albert, 1986; Torrance, 1995). Actual creative behavior probably requires both divergent and convergent thinking (Cropley, 2006; Runco, 2013; Runco & Acar, 2012), and the correlation between SBA and GPA might depart from zero, but for the sake of discriminant validity, it should be small.

If the SBA method is in fact strongly correlated with the traditional method of scoring DT, there may be instances where the SBA method can be used instead of the traditional method. After all, the SBA method provides immediate scores, without judges or raters, so inter-rater reliability is not an issue. The benefits of the SBA method would be especially clear when there are large samples of examinees and when the DT testing is online (e.g., mTurk). The present study is the first to compare a large SBA with the traditional method for scoring DT.

## Method

### Participants and Data Collection

The sample consisted of 250 participants (107 female, 141 male, 1 preferred not to disclose, 1 didn’t answer). The mean age of participants was 33.65 years (*SD* = 10.97), and the average grade point average (GPA) was 3.36 on 4-point scale (*SD* = 0.47). A computerized online system (http://mturk.com) was employed to randomly select participants, who were then prompted to complete provided tasks and the survey. Participants were paid $1.5. Only participants with English as a primary language were accepted.

### Task Description and Testing Procedure

The *Many Uses* test was used to assess divergent thinking (www.creativitytestingservices). This is very much like the Alternate Uses test of Guilford (1968) and the Uses test from Wallach and Kogan (1965). It contained three items (i.e., “toothbrush,” “tire,” “spoon,”) which were presented one at a time. It was given to participants without any limitations on response time or output. Participants were told to type in as many ideas as they could and to take their time. Instructions were paraphrased from earlier research on DT. A computerized online system for creativity assessment called *Creativity Index Testing* (http://cit.sparcit.com) was employed to administer the DT test. After finishing the DT test, participants were given a survey asking about age, gender, and GPA.

### DT Scoring

Responses of three *Many Uses items* were scored using the semantics-based algorithmic (SBA) scoring method, described earlier, and the standard DT scoring method from the *Runco Creativity Assessment Battery* (rCAB; 2011). Descriptions of scores, generated by both methods, are provided below.

#### Traditional DT Scores

The fluency score was computed as the number of answers given to each DT task. The standard DT flexibility score was computed following the method of Runco (1985): Each idea was assigned to an a priori conceptual category (one set of categories for each task) and the score calculated as the number of categories used by the individual. This method has demonstrated good inter-rater and inter-item reliability in numerous investigations (see Runco, 2013). The traditional DT originality score was computed from the statistical infrequency of an answer within the pool of answers. If an idea was unique, it got 100pts. If an idea was given three times, it got (100/3=) 33.3pts. If an idea was given hundred times, it got (100/100=) 1pt.

#### SBA Fluency Score

The SBA fluency score was computed from the number of answers given by the individual. This coincides with standardized DT fluency score. For example, if a participant gave five uses of a “tire,” his or her SBA fluency score was five. As is typical, the quality of the ideas is ignored when scoring fluency. Fluency is just a measure of ideational productivity.

#### SBA Flexibility Score

The SBA flexibility score was computed in the following manner. For each item response, which may consist of one or more discrete ideas, the number of categories into which these answers fall is determined. Each response is analyzed in its entirety, but associations with particular discrete parts of each (e.g., single words) are recognized and processed. The number of flexibility categories is literally digitally computed, which is exactly why this is an algorithmic method. The semantic statistic for any pair of ideas is an algorithmic statistic that reflects semantic similarity between two answers. This method has been used successfully by Acar and Runco (Acar & Runco, 2014), though they relied on three semantic networks when calculating semantic statistics. The system used in the present research utilized 12 semantic networks.

#### SBA Item Originality Score

The SBA originality (SBAIRO) score was computed as an average of all semantic association statistics. These statistics capture how far apart ideas given by any one individual are in the semantic networks. The algorithmic originality score was adjusted by the idea association frequency rate (i.e., frequency of usage in Wikipedia [www.wikipedia.com, 2014]). This adjustment is much like traditional DT scoring where an idea is scored based on frequency of occurrence, which is why the SBA originality index represents a kind of originality. SBAIRO score cannot be computed if the item is non-verbal because it requires a verbal starting point as well as a verbal response, and nonverbal tests do not have verbal starting points. They have figural or visual starting points. This is why the *Many Uses* test was chosen for the present research. It has very clear verbal starting points (i.e., the stimulus object, such as “toothbrush,” “tire,” or “spoon”). The *Many Uses* test is also quite similar to *Alternate Uses* and other uses tests from other DT batteries, including that of Guilford (1968) and Torrance (1995). The MUT only differs from AUT in the objects named in the instructions. Here used tire, toothbrush, and spoon were used instead of Guilford’s (1968) “list uses for a brick” or Wallach and Kogan’s (1965) “list uses for a shoe.” Thus very similar results would be expected from AUT and MUT, though of course that is an empirical question (for future research).

### Analyses

First, the reliability of all computed scores was analyzed. Next, associations between SBA and traditional DT scoring methods were analyzed with correlational methods. Then, analyses were conducted to understand correlations of SBA scores with GPA score and response times.

## Results

### Reliability

Descriptive statistics for all scores are shown in Table 1. Standardized DT and SBA scores yielded satisfactory reliability alpha coefficients, as shown in Table 2. Alphas of SBA scores were slightly lower than that of standardized DT scores, but still at adequate levels. And as more data are processed over time, the underlying semantic statistics of SBA method are expected to become more robust, which will no doubt lead to improved reliability of the SBA scores.

**Table 1:** Descriptive Statistics

DT Item	Index	Min	Max	*M*	*SD*
Toothbrush					
	Fluency	1.00	23	4.14	2.73
	DT-Flexibility	1.00	9	2.56	1.17
	DT-Originality	1.22	100	55.50	28.89
	SBAF	1.00	7	2.40	0.84
	SBAIRO	60	49034.55	1907.64	4020.86
Tire					
	Fluency	1.00	11.00	4.07	1.95
	DT-Flexibility	1.00	7.00	2.82	1.24
	DT-Originality	3.90	100	73.55	24.32
	SBAF	1.00	5.00	2.50	0.83
	SFAIRO	171	73675.02	6217.02	9354.99
Spoon					
	Fluency	1.00	13.00	4.24	2.13
	DT-Flexibility	1.00	6.00	2.49	1.09
	DT-Originality	2.08	100	62.28	26.94
	SBAF	1.00	5.00	2.57	0.84
	SBAIRO	88.33	53697.93	4246.37	8212.29

*Note.* Fluency = total number of ideas; DT-Flexibility = traditional Flexibility score; DT-Orig = traditional Originality score; SBAF = Semantic based algorithm Flexibility; SBAIRO = Semantic based algorithmic Originality score.

The standardized DT originality score was less reliable compared than the other scores, perhaps reflecting its sensitivity to sample sizes. Indeed, this score is based on infrequency – a participant’s performance relative to others in the given sample – hence it can vary greatly depending on the composition of the group. Still, the alphas indicate an acceptable level of inter-item reliability, even for originality. Also, the use of such “local norms” (that is, only comparing ideas to others who took exactly the same tests relying on exactly the same procedure) has many advantages, most of important of which is that ideas from one participant are not compared to much older responses from very different normative samples. The use of local norms is very common in DT testing (Runco, 1991, 2013; Wallach & Kogan, 1965).

**Table 2:** Alpha (α) Inter-item Reliability Coefficients

	Raw α	Standardized α
Fluency	.77	.79
DT-Flexibility	.75	.75
DT-Originality	.62	.62
SBAF	.70	.70
SBAIRO	.71	.76

*Note.* Fluency = total number of ideas; DT-Flexibility = traditional Flexibility score; DT-Orig = traditional Originality score; SBAF = Semantic based algorithm Flexibility; SBAIRO = Semantic based algorithmic Originality score.

### SBA Scores vs DT Scores

The Pearson’s product-moment correlation coefficients between the SBA scores and standardized DT scores are provided in Table 3. The first important observation from Table 3 is a significant correlation between fluency and both the traditional DT flexibility and SBA flexibility scores (*r*s = .79, .86, respectively, *p*s < .0001). Indeed, the more the person ideates, the more likely he or she will diverge and find new angles, thus increasing the diversity of generated ideas.

**Table 3:** Bivariate Correlations Between All DT Indices

Measure	1	2	3	4	5
1. Fluency	–				
2. DT-Flexibility	.79	–			
3. DT-Originality	.31	.31	–		
4. SBAF	.86	.74	.33	–	
5. SBAIRO	-.04	-.03	.36	-.01	–

*Note.* Fluency = total number of ideas; DT-Flexibility = traditional Flexibility score; DT-Orig = traditional Originality score; SBAF = Semantic based algorithm Flexibility; SBAIRO = Semantic based algorithmic Originality score.

Turning to the hypotheses, the analyses confirmed that the SBA flexibility score was correlated to the DT flexibility score at *r* = .74 (*p* < .0001), providing strong evidence for the concurrent validity of the SBA flexibility score. This result provides a level of confidence, needed for the use of SBA flexibility scores instead of standardized DT flexibility scores, specifically in cases of mid- and large-scale studies, where the manual computation of DT flexibility scores is not feasible.

Also interesting was that the other SBA scores showed small, but noticeable correlations (*r*s = .33 and .36, respectively, *p*s < .0001) with the traditional DT originality score. One explanation of this is the statistical nature of all three scores. It can be hypothesized that if one had an opportunity to collect an infinitely large number of responses, the DT originality score would have converged to account for a significant portion of information carried by semantic association characteristics. This presents a very interesting direction for future research, but it is beyond the scope of the present investigation.

The SBA item-response originality score demonstrated an independent nature, as the only noticeable correlation it had was with DT originality score (*r* = .36, *p* < .0001), which was already discussed. The correlation between SBA flexibility and SBA originality scores was non-significant (*r* = -.01), which indicates that the two SBA scores seem to measure two independent aspects of divergent thinking.

### SBA Scores vs GPA

The product moment correlation between SBA flexibility and GPA scores for all participants was non-significant (*r* = .20). However, it was observed that for participants with a GPA score less than 3.0, the correlation increased to *r* = .26 (*p* = .001). All other sufficiently large groupings failed to uncover any significant correlations. The correlation between SBA originality and GPA scores was also non-significant (*r* = -.06), and no sufficiently large groupings (by GPA level) yielded significant correlations.

The correlation of GPA with traditional DT flexibility and DT originality were *r* = .08 and *r* = -.06, but non-significant.

### SBA Scores vs Response Time

The product moment correlation between SBA flexibility scores and the amount of time each participant spent completing the DT test (consisted of 3 Many Uses items) was significant (*r* = .46, *p* < .0001). However, further analysis revealed that for participants who spent at least 178 seconds completing a given DT test, such a correlation diminished (*r* = .24) and remained non-significant for other time ranges above the given threshold. For SBA originality scores, the correlation with corresponding response times was non-significant (*r* = .07), and remained non-significant for all sufficiently large time ranges, suggesting that the originality of the responses was independent of the time spent by participants. The correlation of response time to traditional DT flexibility and DT originality scores were *r* = .45 and *r* = .38, respectively.

## Discussion

These results support the construct validation of the SBA DT scores. Indeed, the construct validation was supported both by the inter-index correlations (e.g., SBA flexibility with traditional flexibility) as well as the low correlations with GPA. Certainly additional research should be carried out with other measures of both DT and something like IQ. Still, at present it appears that only a minimal level of general knowledge (in this case represented by GPA) is required to produce ideas on this particular DT test. Quite a bit of previous research has used the same method as used here, with GPA used as an estimate of general knowledge (e.g., Runco & Smith, 1992). Earlier research has also shown that online testing, such as that used here, provides results that are quite similar to face-to-face testing (Hass, 2015).

The moderate correlations among DT indices are noteworthy given the debate over the use of fluency alone when assessing creative potential. Various investigations have found high correlations between fluency and originality and flexibility, and one interpretation was that this could be used to justify relying on fluency alone. The correlations of the present investigation support the use of a DT profile rather than fluency alone. This is especially true of the fluency-originality correlations, which were quite low (.3 and -.04). Admittedly the suggestion of relying on fluency alone was already questionable, given (a) theories of DT, which include various dimensions and not just fluency (Guilford, 1968; Torrance, 1995), (b) psychometric evidence that the variance attributable to originality or flexibility remains reliable even when the overlap with fluency is covaried (Runco & Albert, 1985), (c) additional data showing that explicit instructions can alter originality and flexibility without changing fluency (Runco & Okuda, 1991), and (d) theories of creativity which emphasize originality (and not fluency).

The correlation between SBA flexibility and SBA originality scores was non-significant (*r* = -.01), which indicates that the two SBA scores seem to measure two independent aspects of divergent thinking. That might come as a surprise, at least given that earlier investigations often find them more highly correlated. Then again, theory suggests that they should be relatively independent, as uncovered here. The fact that the present findings are entirely in line with creativity theory is laudable, even if the flexibility-originality correlation reported here was different (and better) than that reported in previous research.

Interestingly, the two flexibility estimates, one from the computer scoring system and one from the traditional scoring system, were in good agreement (i.e., correlated), but the two originality estimates (computer and traditional) were not related very much at all. This is actually what was expected. That was because both the computer and traditional scoring of flexibility rely on semantic categories, so they certainly should be correlated. Even the traditional system for flexibility uses semantic categories, though the assignment of ideas to particular categories is a human decision, while in the case of the algorithmic system the determination of categories is computerized (i.e., based on comparisons with semantic networks). The good agreement of computer and traditional flexibility was thus expected because both systems rely on semantic categories. The computer and traditional originality indices, on the other hand, were each based on different norms and different logic. The computer originality score was based on semantic distance. This is really all a computer can do–compare ideas given by examinees with existing data, and in particular with semantic networks (e.g., WordNet or Word Association Network). The traditional originality score, on the other hand, does not compare ideas given by examinees with any existing norms. It compared ideas given by examinees with ideas given by other examinees! Thus the low agreement between the computer and the traditional originality indices is not at all surprising.

It does leave us with a question, namely, which is the better originality score, computerized/algorithmic or traditional/human. Very likely, the traditional one is the best choice. That is because it is tied to theories of creativity (Guilford, 1968; Runco & Acar, 2012; Torrance, 1995) where originality is defined as thinking in a novel or unique fashion (i.e., unlike other people). That being said, the ideal may be to use both originality scores, though it might be a good idea to interpret the computer originality index as something like *semantic distance*. The semantic distance index may not replace the traditional originality score, but it may prove to provide useful additional information. And like the computer flexibility score, the computer “originality score” (i.e., semantic distance) is cost efficient. Scoring requires little or no human time to be invested.

There is a larger issue, in addition to cost-efficiency of scoring methods. This larger issue arose when questions were directed at the traditional originality score, the crux being that originality scores are “sample dependent” because points are given by comparing one person’s ideas only with other examinees who took the test(s) at the same time. This question is easily refuted. In actuality, the so-called sample dependency of traditional originality is a virtue or strength rather than a problem. That is because divergent thinking tests have only moderate generalizability (Runco, Abdulla, & Paek, in press) and they are much more sensitive to testing conditions than academic and intelligence tests (Runco, 2013; Wallach & Kogan, 1965). For these reasons it is not fair to compare one person’s ideas to people who took different DT tests or people who took them under different testing conditions. The only fair comparison, for calculating uniqueness and originality, is between one examinee and everyone else in the same sample (i.e., the others who took the test under the same conditions). In short, there are advantages to using “local norms” for calculating the traditional originality scores. Originality could be based on judgments instead of objective novelty, but differences among judges (e.g., Runco, McCarthy, & Svensen, 1994; Runco & Smith, 1992) indicate that this would create all kinds of additional problems.

Even before data were collected it was obvious that the associative nature of networks (i.e., their quantifying how frequently words are associated in some corpus) implies that they are most useful for the understanding of ideational flexibility and less informative about ideational originality. This is not a limitation, however, even though it is originality that is always included in definitions of creativity (for a review, see Runco & Jaeger, 2012). We say that because flexibility is recognized in the more comprehensive theories of divergent thinking (Guilford, 1968; Torrance, 1995), even if it is not always a part of empirical investigation. As a matter of fact flexibility should be included more often, given that it is important for adaptability (Flach, 1990) and allows individuals to avoid the rigidity and conceptual ruts that so often derail problem solving. All of this makes the findings presented here about flexibility that much more important.

The correlations of response time to traditional DT flexibility and DT originality scores were mildly disconcerting. Then again, even the largest of these indicated approximately 20% of the variance was explained by time on task. This is not unreasonable if, as previous research suggested, time on task represents the contribution of intrinsic motivation (Plucker, Runco, & Lim, 2006). The logic here is that, given a choice, an examinee will invests more time only when interested in the tasks at hand. Still, additional research should investigate the relationship of time with DT. The present results suggested that there might be a curvilinear relationship, which in turn implies that there is an optimal amount of time for DT. Research on this question would be very practical, given that it would indicate if there is an optimal amount for testing. Other practical implications include cost-efficiency. As noted above, the algorithmic method using semantic networks is highly cost efficient in that it is immediate and requires no manual computation. It appears that it will lead to similar decisions as those reached with a traditional scoring method. The present results were based on one moderately sized sample, and one test of DT, but results are encouraging and indicate that additional research is warranted.

## References

[r1] Acar S. Runco M. A. (2014). Assessing associative distance among ideas elicited by tests of divergent thinking. Creativity Research Journal, 26, 229–238. 10.1080/10400419.2014.901095

[r2] Acar S. Runco M. A. (2015). Thinking in multiple directions: Hyperspace categories in divergent thinking. Psychology of Aesthetics, Creativity, and the Arts, 9, 41–53. 10.1037/a0038501

[r3] Cropley A. J. (2006). In praise of convergent thinking. Creativity Research Journal, 18, 391–404. 10.1207/s15326934crj1803_13

[r4] Flach F. (1990). Disorders of the pathways involved in the creative process. Creativity Research Journal, 3, 158–165. 10.1080/10400419009534349

[r5] Guilford, J. P. (1968). *Creativity, intelligence, and their educational implications*. San Diego, CA, USA: EDITS/ Robert Knapp.

[r6] Hass R. W. (2015). Feasibility of online divergent thinking assessment. Computers in Human Behavior, 46, 85–93. 10.1016/j.chb.2014.12.056

[r7] Hocevar D. (1979). A comparison of statistical infrequency and subjective judgment as criteria in the measurement of originality. Journal of Personality Assessment, 43, 297–299. 10.1207/s15327752jpa4303_13 16367009

[r8] Hocevar, D., & Bachelor, P. (1989). A taxonomy and critique of measurements used in the study of creativity. In J. A. Glover, R. R. Ronning, & C. R. Reynolds (Eds.), *Handbook of creativity: Perspectives on individual differences* (pp. 53-75). New York, NY, USA: Plenum Press.

[r9] Hocevar D. Michael W. B. (1979). The effects of scoring formulas on the discriminant validity of tests of divergent thinking. Educational and Psychological Measurement, 39, 917–921. doi:. 10.1177/001316447903900427

[r10] Kim K. H. (2005). Can only intelligent people be creative? A meta-analysis. Journal of Secondary Gifted Education, 16, 57–66.

[r11] Milgram, R. M. (1990). Creativity: An idea whose time has come and gone? In M. A. Runco & R. S. Albert (Eds.), *Theories of creativity* (pp. 215-233). Newbury Park, CA, USA: Sage.

[r12] Plucker J. A. Runco M. A. Lim W. (2006). Predicting ideational behavior from divergent thinking and discretionary time on task. Creativity Research Journal, 18, 55–63. 10.1207/s15326934crj1801_7

[r13] Rapp D. N. Samuel A. G. (2002). A reason to rhyme: Phonological and semantic influences on lexical access. Journal of Experimental Psychology: Learning, Memory, and Cognition, 28, 564–571. 10.1037/0278-7393.28.3.564 12018508

[r14] Ruge G. (1992). Experiments on linguistically-based term associations. Information Processing & Management, 28, 317–332. 10.1016/0306-4573(92)90078-E

[r15] Runco M. A. (1985). Reliability and convergent validity of ideational flexibility as a function of academic achievement. Perceptual and Motor Skills, 61, 1075–1081. 10.2466/pms.1985.61.3f.1075

[r16] Runco M. A. (1986). Flexibility and originality in children's divergent thinking. The Journal of Psychology, 120, 345–352. 10.1080/00223980.1986.9712632

[r17] Runco, M. A. (Ed.). (1991). *Divergent thinking*. Norwood, NJ, USA: Ablex Publishing Corporation.

[r18] Runco, M. A. (Ed.). (2013). *Divergent thinking and creative potential* (Vol. 2). Cresskill, NJ, USA: Hampton Press.

[r19] Runco M. A. Abdulla A. M. Paek S.-H. (in press). Which test of divergent thinking is best? *Creativity: Theories-Research-Applications*.

[r20] Runco M. A. Acar S. (2012). Divergent thinking as an indicator of creative potential. Creativity Research Journal, 24, 66–75. 10.1080/10400419.2012.652929

[r21] Runco M. A. Albert R. S. (1985). The reliability and validity of ideational originality in the divergent thinking of academically gifted and nongifted children. Educational and Psychological Measurement, 45, 483–501.

[r22] Runco M. A. Albert R. S. (1986). The threshold hypothesis regarding creativity and intelligence: An empirical test with gifted and nongifted children. Creative Child and Adult Quarterly, 11, 212–218.

[r23] Runco M. A. Jaeger G. J. (2012). The standard definition of creativity. Creativity Research Journal, 24, 92–96. 10.1080/10400419.2012.650092

[r24] Runco M. A. McCarthy K. A. Svensen E. (1994). Judgments of the creativity of artwork from students and professional artists. The Journal of Psychology, 128, 23–31. 10.1080/00223980.1994.9712708

[r25] Runco M. A. Okuda S. M. (1991). The instructional enhancement of the flexibility and originality scores of divergent thinking tests. Applied Cognitive Psychology, 5, 435–441. 10.1002/acp.2350050505

[r26] Runco M. A. Okuda S. M. Thurston B. J. (1987). The psychometric properties of four systems for scoring divergent thinking tests. Journal of Psychoeducational Assessment, 5, 149–156. 10.1177/073428298700500206

[r27] Runco M. A. Smith W. R. (1992). Interpersonal and intrapersonal evaluations of creative ideas. Personality and Individual Differences, 13, 295–302. 10.1016/0191-8869(92)90105-X

[r28] Sowa, J. (1991). *Principles of Semantic networks: Explorations in the representation of knowledge*. San Mateo, CA, USA: Morgan Kaufman Publishers.

[r29] Torrance, E. P. (1995). *Why fly?* Cresskill, NJ, USA: Hampton Press.

[r30] Wallach, M. A., & Kogan, N. (1965). *Modes of thinking in young children: A study of the creativity-intelligence distinction*. New York, NY, USA: Holt, Reinhart, & Winston.

